# In-hospital mortality from severe COVID-19 in a tertiary care center in Mexico City; causes of death, risk factors and the impact of hospital saturation

**DOI:** 10.1371/journal.pone.0245772

**Published:** 2021-02-03

**Authors:** Antonio Olivas-Martínez, José Luis Cárdenas-Fragoso, José Víctor Jiménez, Oscar Arturo Lozano-Cruz, Edgar Ortiz-Brizuela, Víctor Hugo Tovar-Méndez, Carla Medrano-Borromeo, Alejandra Martínez-Valenzuela, Carla Marina Román-Montes, Bernardo Martínez-Guerra, María Fernanda González-Lara, Thierry Hernandez-Gilsoul, Alfonso Gulias Herrero, Karla María Tamez-Flores, Eric Ochoa-Hein, Alfredo Ponce-de-León, Arturo Galindo-Fraga, David Kershenobich-Stalnikowitz, José Sifuentes-Osornio

**Affiliations:** 1 Department of Medicine, Instituto Nacional de Ciencias Médicas y Nutrición Salvador Zubirán, Mexico City, Mexico; 2 Department of Biostatistics, University of Washington, Seattle, WA, United States of America; 3 Department of Infectious Diseases, Instituto Nacional de Ciencias Médicas y Nutrición Salvador Zubirán, Mexico City, Mexico; 4 Emergency Department, Instituto Nacional de Ciencias Médicas y Nutrición Salvador Zubirán, Mexico City, Mexico; 5 Department of Epidemiology, Instituto Nacional de Ciencias Médicas y Nutrición Salvador Zubirán, Mexico City, Mexico; 6 General Director´s Office, Instituto Nacional de Ciencias Médicas y Nutrición Salvador Zubirán, Mexico City, Mexico; Azienda Ospedaliero Universitaria Careggi, ITALY

## Abstract

**Background:**

As the severe acute respiratory syndrome coronavirus 2 (SARS-CoV-2) pandemic has remained in Latin America, Mexico has become the third country with the highest death rate worldwide. Data regarding in-hospital mortality and its risk factors, as well as the impact of hospital overcrowding in Latin America has not been thoroughly explored.

**Methods and findings:**

In this prospective cohort study, we enrolled consecutive adult patients hospitalized with severe confirmed COVID-19 pneumonia at a SARS-CoV-2 referral center in Mexico City from February 26th, 2020, to June 5th, 2020. A total of 800 patients were admitted with confirmed diagnosis, mean age was 51.9 *±* 13.9 years, 61% were males, 85% were either obese or overweight, 30% had hypertension and 26% type 2 diabetes. From those 800, 559 recovered (69.9%) and 241 died (30.1%). Among survivors, 101 (18%) received invasive mechanical ventilation (IMV) and 458 (82%) were managed outside the intensive care unit (ICU); mortality in the ICU was 49%. From the non-survivors, 45.6% (n = 110) did not receive full support due to lack of ICU bed availability. Within this subgroup the main cause of death was acute respiratory distress syndrome (ARDS) in 95% of the cases, whereas among the non-survivors who received full (n = 105) support the main cause of death was septic shock (45%) followed by ARDS (29%). The main risk factors associated with in-hospital death were male sex (RR 2.05, 95% CI 1.34–3.12), obesity (RR 1.62, 95% CI 1.14–2.32)—in particular morbid obesity (RR 3.38, 95%CI 1.63–7.00)—and oxygen saturation < 80% on admission (RR 4.8, 95%CI 3.26–7.31).

**Conclusions:**

In this study we found similar in-hospital and ICU mortality, as well as risk factors for mortality, compared to previous reports. However, 45% of the patients who did not survive justified admission to ICU but did not receive IMV / ICU care due to the unavailability of ICU beds. Furthermore, mortality rate over time was mainly due to the availability of ICU beds, indirectly suggesting that overcrowding was one of the main factors that contributed to hospital mortality.

## Introduction

As the severe acute respiratory syndrome coronavirus 2 (SARS-CoV-2) pandemic has spread throughout the globe, it has currently remained in Latin America. As on September 3rd, 2020, Mexico has become the seventh nation with confirmed cases and the third regarding SARS-CoV-2 related deaths worldwide, lagging only Brazil as the second country with the highest death toll worldwide [1]. Current reports have shown a mortality rate ranging from 8 to 21% in patients hospitalized for SARS-CoV-2 pneumonia, and up to 16 to 78% in those requiring ICU admission [2–5]. In the case of Latin America, a recent report from Honduras showed a crude in-hospital mortality of 39% and as high as 72% in mechanically ventilated patients [6].

It has been reported that socioeconomic factors such as higher poverty rates, high public transportation use, lack of health insurance, poor level of formal education as well as overcrowding housing (and other factors that preclude social distancing and precautionary measures) are associated with an increased rate of in-hospital mortality [7–10]. Besides the unfavorable socioeconomic landscape of Mexico, the high rates of hypertension, type 2 diabetes and obesity pose an imminent threat for in-hospital survival [11]. In addition, health care system associated factors, particularly the high requirements for intensive care unit (ICU) beds reported during this pandemic [12] might have an essential role regarding in-hospital mortality. To face this onslaught, Mexico’s ICU bed availability per 100,000 habitants approaches 1.5 [13], which represents half of those initially contemplated in China, around 10% of those in Italy and between 0.04–0.05% of Germany and USA’s total capacity, respectively [14–16]. This high demand for ICU beds might lead to a delay in ICU admission, which is a well-known, time-dependent factor associated with increased mortality [17]. Besides ICU saturation, the effect of both in-hospital and emergency department overcrowding are associated with unfavorable outcomes and might have an impact in major outcomes (mortality) in the circumstances the country faces [18]. In this study, we aimed to describe the in-hospital mortality in adult patients with confirmed SARS-CoV-2 pneumonia as well as risk factors associated with mortality in those who received the standard of care in a tertiary care center in Mexico City. The impact of hospital overcrowding was explored as well.

## Material and methods

### Study design

In this prospective cohort study, we enrolled all consecutive adult patients hospitalized with severe confirmed COVID-19 pneumonia at a tertiary care center in Mexico City from February 26th, 2020, to June 5th, 2020. The hospital was reconverted to a COVID-19 reference center on March 16th 2020, 96 beds were used for hospital wards, the ICU bed capacity was expanded from 14 to 42 beds (all of them for invasive mechanical ventilation, IMV) and 20 beds were used for intermediate care of critically ill non-intubated patients in the emergency department. The process of patient selection for ICU care was based on bed availability.

All patients included in this cohort had a positive real-time reverse transcription-polymerase chain reaction (PCR) either from a naso/oropharyngeal swab or from a tracheal aspirate by a procedure previously described [17], chest computed tomography scan compatible with diagnosis of COVID-19 pneumonia, routine blood workup (including complete blood count, inflammatory markers, metabolic panel and arterial blood gas analysis) and required hospital admission due to hypoxemia. Clinical outcomes were monitored until August 1st, 2020 (the final date of follow-up) through the institutional electronic medical records. This study was approved by the Institutional Review Board (Comité de Investigación and Comité de Ética en Investigación, reference number 3333) and informed consent was waived due to the minimal risk characteristics of an observational study. A detailed description of the setting, molecular diagnostic procedures and data collection has been previously described [18].

### Data collection

Data regarding clinical, demographic, radiologic and laboratory findings were extracted from the institutional electronic medical records by two physicians and reviewed by a third one for data accuracy. Clinical outcomes were followed and registered until August 1st, 2020.

#### Definitions and outcomes

The primary outcome was in-hospital mortality although no time frame was set for this outcome to occur; all the patients analyzed either died in hospital or were discharged home (with or without home oxygen supply). The cause of death was assigned in consensus by three different clinicians (JVJC, JLCF & CMB) under the following definitions: acute respiratory distress syndrome (ARDS) defined according to the 2012 Berlin criteria [19] in intubated patients; patients who died from hypoxemic respiratory failure but were not intubated were assumed to have ARDS even though a peak end expiratory pressure (PEEP) of 5 cm H20 was not administered (non-invasive mechanical ventilation and high flow nasal cannula were not approved by the epidemiology department in the initial months of the pandemic). The diagnosis of acute kidney injury (AKI) was made either with a 0.3 mg/dl elevation of serum creatinine levels or a decrease in urine output under 0.5 ml/kg/hr for > 6 hrs, staging followed the standard KDIGO guidelines criteria for AKI [20]. Multiorgan dysfunction syndrome (MODS) was defined by clinical or laboratory failure of two or more systems as stated by the SOFA score [21]. In addition to the medical cause of death, one of the following classifications was given in a case by case basis: 1 = directly attributed to SARS-CoV-2 infection (ARDS, septic shock or MODS without an additional nosocomial or opportunistic infection and acute pulmonary embolism); 2 = indirectly attributed to SARS—CoV-2 infection (septic shock or MODS because of nosocomial or opportunistic infections -microbiological evidence was required in this category-), arrhythmias either triggered or directly caused as a result of the critical state or life support (for instance vasopressor use, hemodialysis), precipitated acute coronary syndromes or complications directly attributed to standard management of SARS-CoV-2 pneumonia (e.g. barotrauma secondary to IMV or central line placement, or upper/lower gastrointestinal bleeding secondary to anticoagulation/thrombolysis); 3 = not attributable to SARS-CoV-2 infection. Immunosuppression was considered if the patient was neutropenic (less than 500 neutrophils), asplenic, with an active malignant disease or under immunosuppressive treatment (prednisone >20mg/day or any other immunosuppressive drugs for at least 30 days). Smoking was considered as current tobacco use.

### Statistical analysis

Numerical variables are summarized in mean and standard deviation or median and interquartile range (IQR) according to the shape of the distribution (symmetric or non-symmetric), categorical variables are presented in frequencies and percentages. The characteristics at admission are described overall and by the following mutually exclusive groups: 1) survivor at discharge, 2) non-survivor with full support, 3) non-survivor without full support (because of lack of bed at the ICU and ventilator), and 4) non-survivor with do not intubate (DNI) or do not resuscitate (DNR) orders elected by the patient. The characteristics at admission were also compared between the first two groups (survivor at discharge versus non-survivor with full support) using a t-student test or a U Mann-Whitney test if numeric or a chi-squared test if categorical.

In relation to mortality, causes of death, its association with COVID-19 and the number of deaths per week are described overall and by the groups defined by the support received. The number of deaths per week are also displayed according to the place of death. In order to illustrate the impact of hospital saturation on mortality over time, the following data are graphically displayed per day: the number of patients attended at triage, the number of admitted patients (both confirmed and non-confirmed SARS-CoV-2 cases), the number of confirmed and non-confirmed SARS-CoV-2 deaths, and the confirmed and non-confirmed SARS-CoV-2 mortality rates. The first order trends for the number of admitted patients and for the number of in-hospital patients over time were estimated using linear splines with one knot on the dates of a clear change of behavior. The confirmed and non-confirmed SARS-CoV-2 mortality rate tendencies over time were assessed with locally weighted scatterplot smoothing (LOWESS) curves superimposed to their corresponding mortality rates values per day. Non-confirmed SARS-CoV-2 patients had pneumonia compatible with COVID-19 but with a negative PCR test.

The analysis of risk factors was performed using the data from patients in the first two groups: alive at discharge and died with full support. The following variables were evaluated as risk factors for mortality: sex, age, body mass index (BMI), obesity, diabetes, hypertension, smoking status, NEWS score and O2 saturation breathing air at admission. In order to report risk ratios for all these variables, numeric variables were categorized into binary variables using the closest integer to its overall mean or standardized cutpoints. For each variable, the unadjusted risk ratio for mortality was estimated using the normal approximation to the logarithm of the risk ratio, meanwhile the adjusted risk ratio was estimated using the Targeted Maximum Likelihood Estimation method with a super learning algorithm to estimate both the exposure and outcome mechanisms [22]. The confounders for adjusting the risk ratio estimate were different for each covariate and were selected according to scientific knowledge. No confounders were considered for age and gender. Statistical analysis was performed using R software version 4.0.2. A two-tailed confidence level of 0.05 was established.

## Results

### Study population

**Fig 1:**
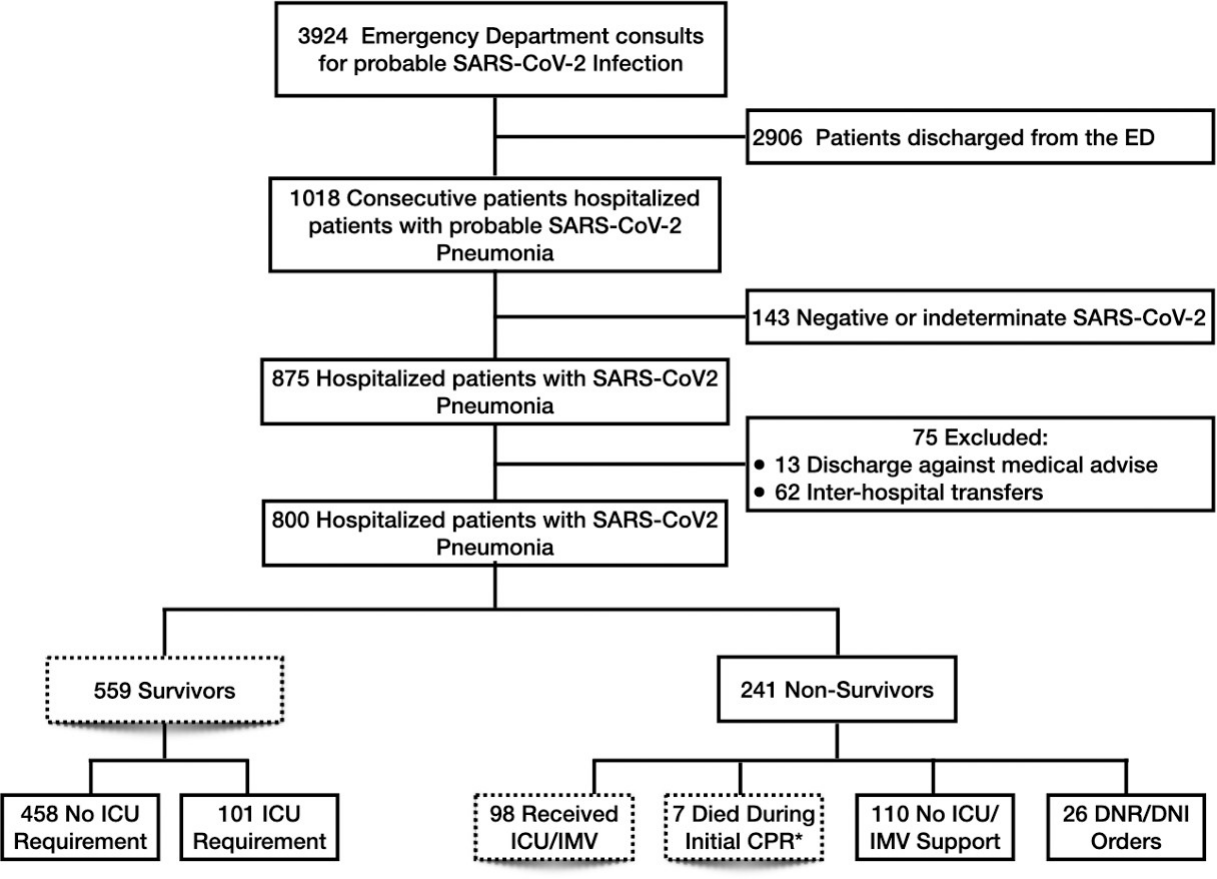
Flowchart of the study design. Only the boxes with dashed lines were included in the risk factors analysis. SARS-CoV-2, severe acute respiratory syndrome coronavirus 2. ICU, intensive care unit. PCR, polymerase chain reaction. DNI/DNR, do not intubate/do not resuscitate. IMV, invasive mechanical ventilation. *These 7 patients died during CPR outside the ICU and did not received IMV.

During the study period, 3924 consecutive patients were evaluated in the emergency department for suspected SARS-CoV-2 infection, of whom 1018 patients (26%) were admitted under the diagnosis of suspected SARS-CoV-2 pneumonia pending on the result of SARS-CoV-2 PCR. Of them, 143 patients (14%) did not meet inclusion criteria due to negative or indeterminate SARS-CoV-2 PCR results and 875 patients (86%) were confirmed SARS-CoV-2 pneumonia cases. Finally, we excluded 62 patients due to inter-hospital transfer and unknown clinical outcome (transfer to another hospital with available ICU beds owing to clinical deterioration) and 13 patients that were discharged against medical advice (Fig 1). To complete the description of the flowchart (Fig 1) in S1 Fig, we described the total number of visits to the emergency department, as well as the number of patients who were not admitted and the total number of hospital admissions. In addition, we included the crude in-hospital mortality of patients with positive SARS-CoV-2 and the in-hospital mortality of patients with negative or indeterminate SARS-CoV-2 results.

The final study population consisted of 800 hospitalized patients with confirmed SARS-CoV-2 pneumonia of whom 559 recovered (69.9%) and 241 died (30.1%) during hospitalization. Among the survivors, 101 (18%) received IMV and 458 were managed either in general hospital wards or intermediate medical care units/emergency department (IMCU/ED) beds. From non-survivor patients, 43.6% (n = 105) received full support (98 received full standard care of treatment including IMV and 7 died during CPR outside ICU areas); 45.6% (n = 110) did not receive full support (they had refractory hypoxemia but were not intubated due to ICU bed availability) and 10.8% (n = 26) were under DNI/DNR orders.

### Population characteristics

Baseline characteristics are summarized in Table 1. Overall, mean age was 51.9 *±* 13.9 years, 61% (n = 488) were males, 46.3% (n = 350) were obese, 38.4% (n = 290) were overweight, mean BMI was 30.3 *±* 5.8 kg/m2, 30% (n = 240) had hypertension and 26% (n = 209) lived with type 2 diabetes. Among the 664 patients who received full support, age was similar in survivors and non-survivors (mean of 48.8 years *±* 12.9 years versus 51.9 *±* 13.9 years, p = 0.054), but mean BMI (mean of 30.2 *± 5*.*4 kg/m2* in survivors and 32.3 *± 7*.*5 kg/m2* in non-survivors, *p = 0*.*007)* and diabetes prevalence (22% in survivors vs 33% in non-survivors, *p = 0*.*015*) were higher in non-survivors. There was no difference among survivors and non-survivors regarding other comorbidities.

**Table 1:** Demographic and clinical characteristics.

Characteristic	N	Overall, N = 800	Alive, N = 559	Died with support, N = 105	Died without full support, N = 110	NI/DNR, N = 26	P-value
Male gender, n (%)	800	488 (61)	325 (58)	80 (76)	71 (65)	12 (46)	**<0.001**
Age, mean (± SD) -years-	800	51.9 (13.9)	48.8 (12.9)	51.3 (11.5)	64.1 (10.7)	70.3 (12.4)	0.054
Weight, mean (± SD) -kg-	764	81.4 (17.4)	81.3 (16.1)	88 (21.9)	77.9 (16.4)	71.1 (16.7)	**0.004**
Height, mean (± SD) -m-	757	1.63 (9.6)	1.64 (9.6)	1.64 (9.2)	1.62 (9.2)	1.58 (8.2)	0.52
BMI, mean (± SD)—kg/m^2^-	756	30.3 (5.8)	30.2 (5.4)	32.3 (7.5)	29.6 (5.3)	28.5 (6)	**0.007**
BMI classification							**0.021**
Normal BMI, n (%)		116 (15.3)	82 (15.7)	10 (9.7)	15 (14.4)	9 (34.6)	
Overweight, n (%)	780	290 (38.4)	201 (38.4)	35 (34)	46 (44.2)	8 (30.8)	
Obesity, n (%)	797	357 (44.8)	245 (44.1)	59 (56.2)	43 (39.1)	10 (38.5)	
-Obese class I, n (%)		223 (29.5)	157 (30)	34 (33)	28 (26.9)	4 (15.4)	
-Obese class II, n (%)		84 (11.1)	58 (11.1)	11 (10.7)	11 (10.6)	4 (15.4)	
-Obese class III, n (%)		43 (5.7)	25 (4.8)	13 (12.6)	4 (3.8)	1 (3.8)	
Healthcare workers, n (%)	799	51 (6.4)	41 (7.3)	7 (6.7)	2 (1.8)	1 (3.8)	0.97
Diabetes, n (%)	800	209 (26)	122 (22)	35 (33)	46 (42)	6 (23)	**0.015**
Hypertension, n (%)	800	240 (30)	146 (26)	32 (30)	50 (45)	12 (46)	0.42
Chronic lung disease, n (%)	800	7 (0.9)	3 (0.5)	1 (1)	3 (2.7)	0 (0)	>0.99
Asthma, n (%)	799	11 (1.4)	8 (1.4)	1 (1)	2 (1.8)	0 (0)	>0.99
Immunosupression, n (%)	800	48 (6)	31 (5.5)	3 (2.9)	11 (10)	3 (12)	0.37
HIV, n (%)	800	10 (1.2)	9 (1.6)	0 (0)	1 (0.9)	0 (0)	0.4
Cardiovascular disease, n (%)	799	37 (4.6)	23 (4.1)	7 (6.7)	4 (3.6)	3 (12)	0.37
Chronic kidney disease, n (%)	800	24 (3)	17 (3)	1 (1)	4 (3.6)	2 (7.7)	0.38
Chronic liver disease, n (%)	797	6 (0.8)	4 (0.7)	1 (1.0)	1 (0.9)	0 (0)	>0.99
Smoking, n (%)	790	112 (14)	84 (15)	11 (10)	16 (15)	1 (3.8)	0.27
Pneumonia seen on CT scan, n (%)	792	780 (98)	550 (98)	99 (97)	107 (100)	24 (100)	0.6
NIH severity scale, n (%)	797						**<0.001**
Moderate		24 (3)	24 (4.3)	0 (0)	0 (0)	0 (0)	
Severe		744 (93)	524 (94)	90 (87)	104 (95)	26 (100)	
Critical		29 (3.6)	10 (1.8)	13 (13)	6 (5.5)	0 (0)	
MuLBSTA, mean (± SD)	785	7.8 (2.6)	7.4 (2.4)	7.8 (2.9)	9.4 (2.5)	9 (3)	0.16
Charlson, mean (± SD)	800	1.5 (1.7)	1.2 (1.6)	1.3 (1.4)	2.7 (1.4)	3.3 (1.5)	0.45
qSOFA, n (%)	790						**<0.001**
0		129 (16)	115 (21)	7 (7)	5 (4.6)	2 (7.7)	
1		579 (73)	402 (72)	76 (76)	84 (77)	17 (65)	
2		76 (9.6)	38 (6.8)	16 (16)	16 (15)	6 (23)	
3		6 (0.8)	0 (0)	1 (1)	4 (3.7)	1 (3.8)	
NEWS, mean (± SD)	786	8.5 (2.2)	8.1 (2.3)	9.2 (1.9)	9.5 (1.7)	9.5 (1.6)	**<0.001**

Abbreviations: SD, standard deviation; BMI, body mass index; DNI, do not intubate; DNR, do not resuscitate.

Clinical evaluation at admission is described in Table 2. The median time from symptoms onset to hospital admission was 8 days (IQR: 6–10). The most common symptoms were cough in 90% (n = 716), fever in 87% (n = 693), dyspnea in 80% (n = 633), malaise in 79% (n = 622) and headache in 74% (n = 583). Regarding the physical examination, average mean arterial pressure was 91.4 *±* 12.3 mm Hg, mean heart rate was 103 *±* 18 bpm, median respiratory rate was 28 bpm (IQR: 22–34) and median O2 saturation breathing air was 84% (IQR: 71–88). When comparing the patients who received full support, dyspnea (75% in survivors versus 90% in non-survivors, *p < 0*.*001*), tachypnea (median of 25 bpm in survivors versus 32 bpm in non-survivors, *p < 0*.*001*), tachycardia (mean of 102.3 bpm in survivors and 106.8 bpm in non-survivors, p = 0.018) and lower oxygen desaturation by pulse oximetry at the moment of hospital admission (86% in survivors vs 71.5% in non-survivors, *p < 0*.*001*) were more frequently observed in patients who did not survived. No difference was found in the remaining clinical or physical examinations findings on admission.

**Table 2:** Signs and symptoms on admission.

Characteristic	N	Overall, N = 800	Alive, N = 559	Died with support, N = 105	Died without full support, N = 110	NI/DNR, N = 26	P-value
Days onset symptom-admission, median (IQR) -days-	800	8 (6, 10)	8.0 (6.0, 10.0)	7.0 (6.0, 9.0)	8 (4.2, 10)	7 (5, 9.8)	0.47
Heart rate, mean (± SD) -bpm-	798	103 (17.7)	102 (17.3)	107 (17.7)	103 (17.7)	100 (22.7)	**0.018**
Mean arterial pressure, mean (± SD) -mmHg	777	91 (12.3)	92 (11.8)	91 (11.9)	90 (14.3)	90 (16.2)	0.54
Respiratory rate, median (IQR) -bpm-	796	28 (22, 34)	25 (22, 30)	32 (25, 40)	32 (28, 40)	33 (26, 40)	**<0.001**
O2 saturation breathing air, median (IQR)—%-	774	84 (71, 88)	86 (80, 89)	71 (51, 81)	68 (51, 81)	65 (55, 70)	**<0.001**
Fever, n (%)	799	693 (87%)	483 (86)	95 (91)	95 (86)	20 (77)	0.22
Cough, n (%)	793	716 (90)	502 (91)	98 (93)	92 (85)	24 (92)	0.48
Headache, n (%)	788	583 (74)	428 (78)	71 (70)	67 (61)	17 (65)	0.1
Dyspnea, n (%)	794	633 (80)	418 (75)	93 (90)	101 (93)	21 (81)	**0.001**
Diarrhea, n (%)	759	235 (31)	172 (32)	28 (28)	29 (28)	6 (24)	0.45
Chest pain, n (%)	757	260 (34)	188 (36)	40 (39)	22 (22)	10 (40)	0.62
Chills, n (%)	752	395 (53)	283 (54)	52 (52)	49 (49)	10 (40)	0.88
Odynophagia, n (%)	782	384 (49)	279 (51)	46 (45)	45 (42)	14 (54)	0.32
Myalgias, n (%)	757	499 (66)	364 (69)	60 (59)	64 (63)	11 (44)	0.084
Arthralgias, n (%)	759	459 (60)	333 (63)	55 (54)	59 (57)	12 (48)	0.14
Malaise, n (%)	791	622 (79)	440 (79)	78 (76)	82 (76)	22 (85)	0.48
Rhinorrhea, n (%)	757	230 (30)	166 (31)	28 (28)	28 (28)	8 (31)	0.59
Vomiting, n (%)	751	97 (13)	70 (13)	14 (14)	13 (13)	0 (0)	0.98
Abdominal pain, n (%)	753	98 (13)	73 (14)	13 (13)	8 (7.9)	4 (16)	0.95
Conjunctivitis, n (%)	751	100 (13)	82 (16)	10 (10)	6 (5.9)	2 (8)	0.19
Cyanosis, n (%)	751	28 (3.7)	12 (2.3)	6 (6)	8 (8)	2 (8)	0.087
Anosmia, n (%)	375	55 (15)	46 (18)	2 (4.9)	6 (9.8)	1 (7.7)	0.064
Dysgeusia, n (%)	336	37 (11)	32 (14)	0 (0)	4 (7.3)	1 (8.3)	**0.03**
Glasgow coma scale < 15 points, n (%)	791	26 (3.3)	7 (1.3)	7 (6.9)	8 (7.3)	4 (15)	**0.001**

Abbreviations: IQR, interquartile range; SD, standard deviation; DNI, do not intubate; DNR, do not resuscitate.

The laboratorial features at admission are presented in Table 3. Overall, inflammatory measures were elevated: median leucocyte count of 8,100 cells/uL (IQR: 5,800–11,300), median neutrophil count of 6.7 x 10^3^ cells/uL (IQR: 4.5 x 10^3^–10.0 x 10^3^), median neutrophil-lymphocyte ratio of 8.6 (IQR: 5.1–15.2), median C-reactive protein level of 14.9 mg/dL (IQR: 8.0–23.2), median D-dimer level of 774 ng/mL (IQR: 462–1191), median ferritin level of 621 ng/mL (IQR: 322–1068), median fibrinogen level of 695 mg/dL (IQR: 544–834) and median LDH level of 384 U/L (IQR: 302–506), whereas the PaO2/FiO2 ratio was low (median of 206, IQR: 125–263). After comparing survivors and patients with full support who died, lower lymphocyte (median of 826 cells/uL in survivors versus 676 cell/uL in non survivors, *p = 0*.*005)*, higher neutrophil counts (median 6.0 x10^3^ cells/uL in survivors versus 8.9 x10^3^ cells/uL in non-survivors, *p = <0*.*001)*, higher neutrophil-to-lymphocyte ratio (median of 7 in survivors versus 12.1 in non-survivors, p *= <0*.*001)*, as well as, increased inflammatory markers such as ferritin (median of 564 ng/ml versus 850 ng/ml, *p = <0*.*001*), C-reactive protein (median of 12.9 g/dl versus 21.4 g/dl, *p = <0*.*001)* and LDH (median of 354 U/L versus 518 U/L, *p = <0*.*001)* levels in addition to increased prothrombotic markers (median D-dimer of 677 ng/ml versus 919 ng/ml, *p = < 0*.*001*; median fibrinogen of 678 mg/dl versus 751 mg/dl, *p = 0*.*005)* were found among non-survivors. Patients who died during hospitalization showed more severity on markers of end-organ dysfunction than those who recovered, manifested as higher creatinine (median of 0.9 mg/dl versus 1.0 mg/dl, *p = 0*.*002)* and blood urea nitrogen levels (median of 13.9 mg/dl versus 17.9 mg/dl, *p = < 0*.*001)*, lower PaO2/FiO2 ratio (median of 230 versus 118, *p = < 0*.*001)* and increased troponin levels (median of 4.6 pg/ml versus 12.4 pg/ml, *p = <0*.*001)*.

**Table 3:** Laboratory values on admission.

Characteristic	N	Overall, N = 800	Alive, N = 559	Died with support, N = 105	Died without full support, N = 110	NI/DNR, N = 26	P-value
Blood urea nitrogen, mean (IQR) -mg/dl-	792	15 (11, 22)	13 (10, 19)	18 (13, 28)	22 (16, 37)	28 (20, 38)	**<0.001**
Creatinine, mean (IQR) -mg/dl-	792	0.9 (0.8, 1.2)	0.9 (0.8, 1.1)	1.0 (0.8, 1.3)	1.1 (0.8, 1.6)	1.1 (1, 1.7)	**0.002**
Total bilirubin, mean (± SD) -mg/dl-	780	0.7 (0.5)	0.7 (0.5)	0.8 (0.8)	0.7 (0.3)	0.8 (0.3)	0.26
ALT, median (IQR) -U/L-	780	37 (24, 56)	37 (24, 57)	42 (29, 58)	34 (23, 50)	31 (24, 50)	0.055
AST, median (IQR) -U/L-	780	44 (31, 64)	41 (29, 59)	53 (42, 77)	53 (35, 70)	42 (35, 66)	**<0.001**
ALP, median (IQR) -U/L-	780	90 (71, 118)	89 (70, 115)	92 (67, 119)	97 (77, 117)	123 (84, 173)	0.89
Albumin, mean (± SD) -g/dl-	779	3.7 (0.5)	3.8 (0.4)	3.4 (0.5)	3.4 (0.5)	3.3 (0.4)	**<0.001**
CRP, median (IQR) -g/dl-	769	14.9 (8, 23.2)	12.9 (6.1, 19.6)	21.4 (14.8, 28.4)	18.7 (13.8, 28.8)	22.1 (17.2, 29)	**<0.001**
Leukocytes, median (IQR) -10^3/uL-	789	8.1 (5.8, 11.3)	7.3 (5.4, 9.97)	10.25 (7.82, 13.95)	10.25 (7.07, 13.32)	11 (8.9, 15)	**<0.001**
Hemoglobin, mean (± SD) -g/dl-	791	15.2 (2.1)	15.2 (2.1)	15.5 (1.9)	14.9 (2)	14 (2.1)	0.21
Platelets, median (IQR) -K/uL-	789	218 (175, 284)	214 (172, 281)	222 (183, 267)	229 (175, 290)	247 (216, 301)	0.56
Absolute lymphocyte count, median (IQR)	786	776 (539, 1045)	826 (598, 1088)	676 (485, 943)	576 (429, 805)	712 (451, 962)	**0.005**
Absolute neutrophil count, median (IQR)	787	6685 (4472, 9998)	6019 (4014, 8471)	8913 (6630, 12062)	8749 (6013, 12337)	9995 (7524, 13604)	**<0.001**
Neutrophil-lymphocyte ratio, median (IQR)	785	8.6 (5.1, 15.2)	7 (4.3, 12.3)	12.1 (7.9, 18.6)	13.7 (9.3, 24.8)	17.1 (11.9, 21.7)	**<0.001**
D-dimer, median (IQR) -ng/mL-	760	774 (461, 1191)	677 (425, 1057)	919 (628, 1398)	1079 (673, 2109)	1401 (1019, 2092)	**<0.001**
CPK, median (IQR) -U/L-	708	111 (60, 239)	103 (57, 227)	165 (96, 392)	104 (57, 226)	112 (66, 174)	**<0.001**
LDH, median (IQR) -U/L-	749	384 (302, 506)	354 (277, 450)	518 (389, 648)	501 (383, 654)	508 (380, 646)	**<0.001**
Fibrinogen, median (IQR) -mg/dl-	670	695 (544, 834)	678 (501, 807)	751 (595, 929)	768 (632, 896)	722 (582, 920)	**0.005**
Troponin I, median (IQR) -pg/ml-	702	5.7 (3.7, 12.6)	4.6 (3.2, 7.2)	12.4 (6.3, 76.9)	15.2 (7.3, 50.4)	44.4 (13.7, 452.9)	**<0.001**
Ferritin, median (IQR) -ng/ml-	759	621 (321, 1068)	565 (262, 948)	850 (461, 1359)	682 (408, 1227)	673 (361, 903)	**<0.001**
pH, median (IQR)	769	7.4 (7.4, 7.5)	7.4 (7.4, 7.5)	7.4 (7.4, 7.5)	7.4 (7.4, 7.5)	7.4 (7.4, 7.4)	**<0.001**
Partial pressure oxygen, median (IQR) -mmHg-	769	63 (53, 78)	63 (54, 78)	63 (52, 80)	62 (52, 78)	58 (49, 70)	0.71
Partial pressure carbon dioxide, median (IQR) -mmHg-	769	31 (28, 34)	32 (29, 34)	31 (28, 36)	30 (27, 349	30 (27, 33)	0.60
Lactate, median (IQR) -mmol/L-	765	1.2 (1, 2)	1.3 (1, 1.6)	1.8 (1.3, 2.5)	2 (1.4, 3.4)	2.5 (1.6, 3.6)	**<0.001**
PaO2/FiO2, median (IQR)	764	206 (125, 2639	230 (163, 273)	118 (86, 189)	152 (94, 221)	106 (82, 132)	**<0.001**

Abbreviations: IQR, interquartile range; SD, standard deviation; DNI, do not intubate; DNR, do not resuscitate; ALT, alanine aminotransferase; AST, aspartate aminotransferase; ALP alkaline phosphatase; CRP, C-reactive protein; CPK, creatine phosphokinase; DHL lactate dehydrogenase; ESR, erythrocyte sedimentation rate. SI conversion factor: To convert creatinine to μmol/L, multiply values by 88.4

In relation to patients who died without receiving full support because of unavailability of critical care beds or DNR/DNI order, they appeared to be older, with lower BMI and lower PaO2/FiO2 ratios than those who received full support. However, the observed markers of inflammation and end-organ dysfunction appeared to be similar (Table 1).

### Hospital attention and mortality over time

From March 16th to June 5th, 3924 subjects were evaluated at triage and exclusively COVID-19 suspected cases assessed since March 20th. Of them, 26% (n = 1018) were admitted, with a global mortality of 27% (n = 278, 241 confirmed COVID-19 cases and 37 non-confirmed cases). Since the beginning of the study the number of admissions increased until April 24th with a linear trend of 0.4 more admissions per day and oscillated around a mean of 15 admissions per day afterwards (S1A Fig). The number of in-hospital patients per day stratified by their vital status (alive versus died on each day) is displayed in (S1B Fig). The count of in-hospital patients increased until April 28th with a linear trend of 3.5 more patients per day and with a linear trend of 0.5 more patients per day subsequently. S1C Fig shows the mortality rates in confirmed and non-confirmed cases with LOWESS curves superimposed. The mortality rate in confirmed cases had an important increase until April 27th, stabilized during the first half of May and began to decrease afterwards. The behavior of mortality rate in non-confirmed cases was similar but with a mortality rate of 2.7% on March 30th, which corresponds to one death in a day with only 37 in-hospital patients, and stabilization after May 5th.

### Description of deaths in confirmed COVID-19 cases

**Fig 2:**
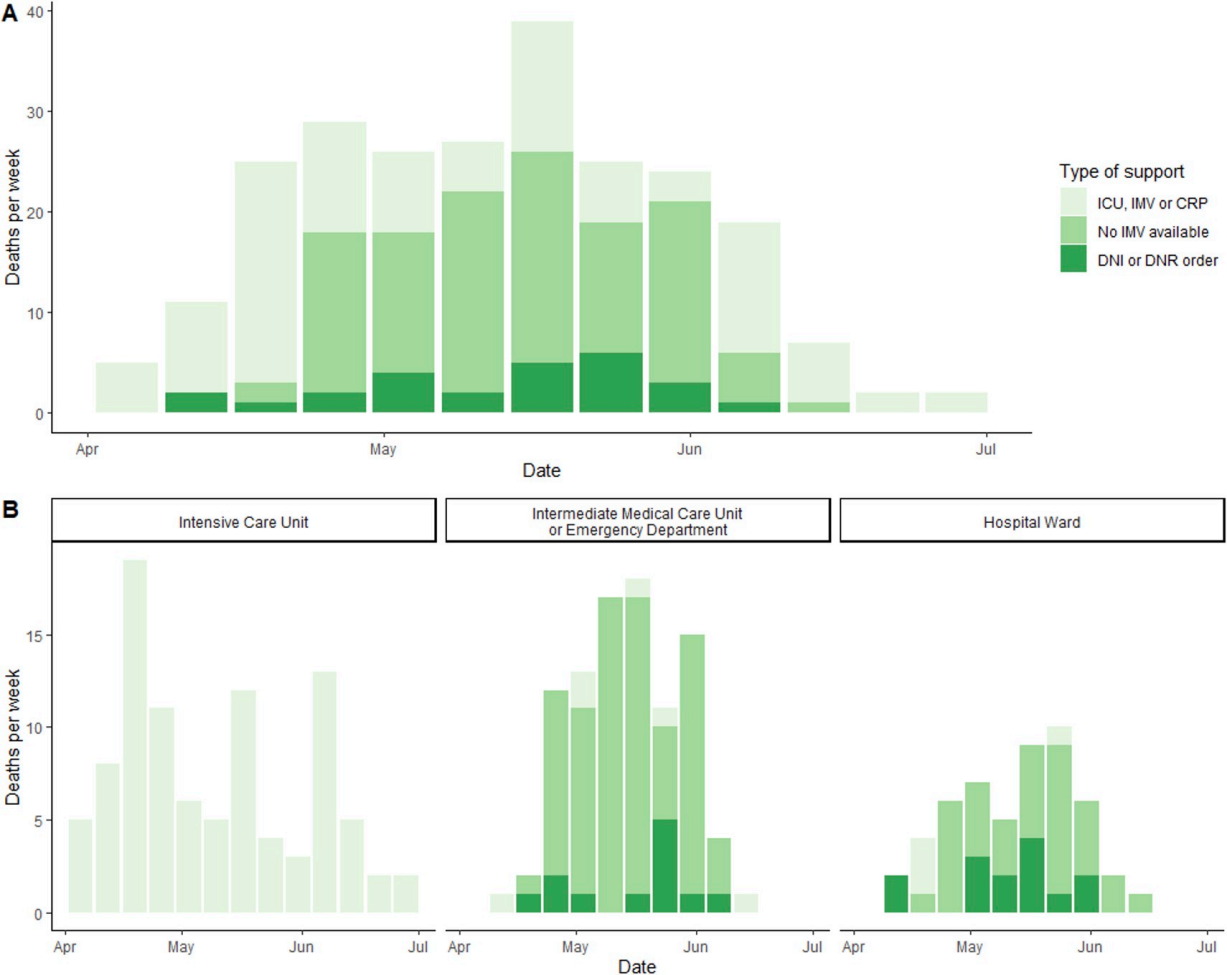
Bar graph. **Panel A:** Timeline of mortality during the course of the study divided by patients who received ICU/IMV/CPR support, those who were limited due to resource availability (NO IMV) and those who decided DNI/DNR status. **Panel B:** Timeline of mortality divided by hospitalization areas.

The distribution of deaths stratified by the support received is shown in Fig 2A. The first death occurred on April 5th and the last one on July 3rd. Deaths in patients without full support began during the third week of April (April 22th), with a substantial increase in the last week of April, which was preserved until the first days of June. Previous information is further stratified by the place of death in Fig 2B. Of the total of 241 deaths, 39.4% (n = 95) occurred in the ICU, 39.0% (n = 94) in the IMCR/ED and the remaining 21.6% (n = 52) in the hospital ward. All deaths that occurred in the ICU received full support and decreased in number over time, whereas most of the patients who died outside the ICU did not receive full support (76 in the IMCU/ED and 34 in the hospital ward). Ten patients who received full support died outside the ICU, 6 in the IMCU/ED (4 during CRP and 2 after UCI discharge from a non-respiratory cause) and 4 in the hospital ward (2 during CRP and 2 after UCI discharge from a non-respiratory cause).

Overall, the main cause of death was known in 239 of the 241 non-survivors and was ARDS in 67%, (n = 159) followed by septic-shock in 19% (n = 45) and MODS in 9.2% (n = 22). Arrhythmias, pulmonary embolism and acute coronary syndromes were rare causes of death in this cohort. We further analyzed the cause of death among patients who were not admitted to the ICU despite invasive mechanical ventilation requirements (due to lack of ICU beds) the vast majority of them died of hypoxic respiratory failure/ARDS (105/110, 95%). In contrast, among non-survivors who were admitted to the ICU the main cause of death was septic shock in 42% (43/105) followed by ARDS in 29% (30/105) and MODS in 18% (19/105). All of the patients who were admitted to the ICU and died (105/105) had respiratory failure, 85% (88/105) hemodynamic failure and 49% (50/105) renal failure; and 85% (88/105) had more than one organ failure. Information regarding causes of death in the group of non-survivors, those patients admitted to ICU and those not admitted into the ICU are described in Table 4, 88% (210/239) of the total deaths were directly attributed to SARS-CoV-2 infection and 12% (29/239) were indirectly associated.

**Table 4:** Causes of death.

Characteristic	N	Overall, N = 241	Died with support, N = 105	Died without full support, N = 110	NI/DNR, N = 26
Predominant terminal organ failure					
Multiorganic, n (%)	239	126 (53)	88 (85)	31 (28)	7 (27)
Hemodynamic, n (%)	239	105 (44)	88 (85)	14 (13)	3 (12)
Renal, n (%)	239	75 (31)	50 (49)	21 (19)	4 (15)
Hepatic, n (%)	239	0 (0)	0 (0)	0 (0)	0 (0)
Hematologic, n (%)	239	7 (2.9)	2 (1.9)	5 (4.5)	0 (0)
Cause of death	239				
Unstable bradycardia, n (%)		1 (0.4)	1 (1.0)	0 (0)	0 (0)
Hypovolemic shock (GI bleeding), n (%)		2 (0.8)	2 (1.9)	0	0
Septic shock, n (%)		45 (19)	43 (42)	2 (1.8)	0 (0)
Super-refractory status epilepticus / Stroke, n (%)		1 (0.4)	1 (1)	0 (0)	0 (0)
STEMI, n (%)		1 (0.4)	1 (1)	0 (0)	0 (0)
Multiorganic failure, n (%)		22 (9.2)	19 (18)	2 (1.8)	1 (3.8)
Airway obstruction, n (%)		2 (0.8)	2 (1.9)	0 (0)	0 (0)
Acute respiratory distress syndrome, n (%)		159 (67)	30 (29)	105 (95)	24 (92)
Pulmonary embolism, n (%)		4 (1.7)	2 (1.9)	1 (0.9)	1 (3.8)
Unstable supraventricular tachycardia, n (%)		2 (0.8)	2 (1.9)	0 (0)	0 (0)
COVID-19 association	239				
Directly associated, n (%)		210 (88)	78 (76)	106 (96)	26 (100)
Indirectly associated, n (%)		29 (12)	25 (24)	4 (3.6)	0 (0)
Unknown		2	2	0	0

Abbreviations: ICU, intensive care unit; IMV, invasive mechanical ventilation; CPR, cardiopulmonary resuscitation; DNI, do not intubate; DNR, do not resuscitate; GI, gastrointestinal; STEMI, ST-elevation myocardial infarction; COVID-19, coronavirus disease 2019

### Risk factors of death

The analysis of the studied risk factors for mortality is summarized in Table 5. We estimated that the risk of in-hospital death was significantly higher in males than in females (RR of 2.05, 95% CI 1.34–3.12, p < 0.001), in obese than in non-obese patients (RR of 1.62, 95% CI 1.14–2.32, p = 0.008), in morbid obese subjects than in those with normal BMI (RR of 3.38, 95% CI1.63–7.00, p = 0.001), in diabetic than non-diabetic ones (RR of 1.47, 95% CI 1.01–2.15, p = 0.046), in patients with a NEWS score ⩾7 as compared to those with less than 7 points at admission (RR of 2.44, 95% CI 1.18–5.03, p = 0.016) and in subjects with ⩾ 80% oxygen saturation than in those with less than 80% oxygen saturation breathing air at admission (RR of 4.88, 95% CI 3.26–7.31, p < 0.001).

**Table 5:** Risk factors associated with mortality.

		Unadjusted estimate			Adjusted estimate		
Gender	Risk	RR	95% CI	P value	aRR	95% CI	P value
Males	20%	2.05	1.34–3.12	**< 0.001**			
Females	9.7%						
**Age**							
≥ 50 yr.	17%	1.11	0.78–1.58	0.56			
< 50 yr.	15%						
**Obesity (BMI ≥ 30 kg/m**^**2**^**)**					Adjusted by age and gender		
Yes	19%	1.42	0.99–2.03	0.054	1.62	1.14–2.32	**0.008**
No	14%						
**BMI Classification**					Adjusted by age and gender		
Normal BMI	11%	Reference			Reference		
Overweight	15%	1.36	0.71–2.64	0.36	1.37	0.72–2.63	0.34
Obese class I	18%	1.64	0.85–3.17	0.14	1.70	0.89–3.21	0.11
Obese class II	16%	1.47	0.66–3.26	0.35	2.02	0.94–4.34	0.073
Obese class III	34%	3.15	1.51–6.55	0.002	3.38	1.63–7.00	**0.001**
**Morbid obesity (BMI ≥ 40 kg/m**^**2**^**)**					Adjusted by age and gender		
Yes	34%	2.24	1.38–3.61	0.001	2.41	1.53–3.81	**< 0.001**
No	15%						
**Diabetes**					Adjusted by age, gender and BMI		
Yes	22%	1.61	1.12–2.32	0.010	1.47	1.01–2.15	**0.046**
No	14%						
**Hypertension**					Adjusted by age, gender and BMI		
Yes	18%	1.20	0.82–1.75	0.35	0.99	0.66–1.50	0.98
No	15%						
**Smoking**					Adjusted by age and gender		
Yes	12%	0.69	0.39–1.25	0.22	0.62	0.35–1.09	0.09
No	17%						
**NEWS Score**					Adjusted by age, gender, BMI, diabetes, hypertension and smoking status		
≥ 7	17%	2.61	1.35–5.04	0.004	2.44	1.18–5.03	**0.016**
< 7	6.7%						
**O2 saturation breathing air**					Adjusted by age, gender, BMI, and smoking status		
< 80	37%	5.83	3.90–8.72	< 0.001	4.88	3.26–7.31	**< 0.001**
≥ 80	6.3%						

Abbreviations: RR, risk ratio; aRR, adjusted risk ratio; CI, confidence interval; BMI, body mass index.

The analyses were performed using data from the 664 patients who were alive at discharge or died with support. There were 38 missing observations for BMI, 5 for smoking, 12 for the NEWS score and 23 for O2 saturation breathing air. Thus, there were 38 missing observations for the adjusted analysis of BMI, diabetes, and hypertension, 52 for the adjusted analysis of the NEWS score and 63 for the one of the O2 saturation breathing air level. Adjusted risk ratios were estimated using Targeted Maximum Likelihood Estimation with a super learning algorithm to estimate both the exposure and outcome mechanisms.

## Discussion

In this prospective cohort study among patients with confirmed COVID-19 pneumonia, attended in a tertiary care center located in Mexico City, in-hospital mortality was 30.1%, and 49.2% in the ICU beds (which were composed of more than 50% enabled ICU beds), both falling within the range described in previous reports (20–40% and 50–70%, respectively) [2–6, 23–25]. It is worth noting that all (199/199) the patients admitted to the ICU required mechanical ventilation, in contrast to other cohorts in which 69–88% of the ICU admission required this modality of ventilatory support [23–25]. It is important to emphasize that until the final date of inclusion for this study, the use of high-flow nasal cannula or non-invasive mechanical ventilation was not approved by the infection control committee (due to the potential risk of aerosolization) and therefore they were not used in ICU nor in general hospital wards.

Although the crude mortality seems to be similar to other cohorts, 45% of the non-survivor patients and 14% of the hospitalized patients who developed critical illness and warranted ICU admission did not received IMV/ICU care due to the lack of ICU bed availability (not only in this hospital but in the whole Metropolitan area of Mexico City). These numbers shed light about the unfortunate but urgent problem of health system saturation and ICU resource rationing during the pandemic. This behavior was similarly observed during the initial surge of the pandemic in both Wuhan [26] and New York City [2] where over 50% of the critically ill patients who required ICU care died in general hospital wards and did not receive IMV due to resource constraints [5]. We speculate that overcrowding of the ICU in this tertiary care center was a main determinant for this phenomenon. The rise in deaths between May and June was mainly driven by patients who did not receive IMV/ICU admission. This indirectly reveals a shortage in ICU beds (as well as hospital overcrowding) therefore a delay in ICU admission [27] which is an independent (as well as proportional for the time of delay) risk factor for ICU mortality [28] and, in the particular case of ARDS, a major factor perpetuating lung injury whenever IMV is imminently indicated (due to patient self-inflicted lung injury) [29]. The decrease in mortality throughout time is mostly impelled by the decrease in deaths among patients who did not receive IMV/ICU admission, although the ICU mortality also decreased over time which we believe might be explained by ICU care improvement and acquisition of expertise in the management of these patients in the enabled ICU areas. We included dexamethasone in the standard of care on June 30, 2020, once a preprint of the RECOVERY study [30] was available and NIH released a formal recommendation [31].

In regard to patient related factors associated with mortality, the findings of this study are similar to previous reports in which increased number of comorbidities, especially diabetes and obesity (the grade of the latter being directly proportional with mortality), male gender as well as increased inflammatory markers and laboratory findings related with organic failure were associated with an increased risk of in-hospital death. In a recent analysis of more than 17 million people and over 10,000 related to COVID-19 deaths in the United Kingdom in which multiple ethnicities were included [32], the risk factors associated with mortality are consistent with ours. Interestingly, in that same study [32], race other than caucasian was associated with an increased risk of death (whether Asian, black or mixed). The findings of this study suggest that previously known risk factors might not differ significantly among the Mexican population except for age. In patients who received full support, age was not associated with mortality. However, in this cohort the mean age was a decade lower compared to previous reports from Europe and North America [2, 12, 32–35]. The same pattern was observed in a recent report from Honduras in which a younger age among hospitalized patients was noted and attributed both to a younger population distribution and a higher prevalence of comorbidities at younger ages. Under these speculations the authors of this study rightfully predicted a similar behavior in other Latin American countries as observed in this cohort [6].

In this study, prevalence of diabetes was nearly twice the known national prevalence (13.7% according to the 2016 National Health and Nutrition Survey [41]), but prevalence of hypertension, overweight and obesity were similar (31.5%, 39.1% and 36.1%, respectively [36–39]). Living with diabetes increased 1.5-fold the risk of dying whereas hypertension was not associated with increased mortality. In relation to the weight, the mortality was 11% in patients with normal weight, 15% in those with overweight, 18% in grade 1 obesity, 16% in grade 2 and 34% in patients with morbid obesity. Moreover, in the analysis of risk factors, we found a 3.4-fold increase in mortality risk in patients with morbid obesity as compared to those with normal BMI after adjusting by age and gender. Hence, we believe that the main burden of weight in in-hospital mortality was due to morbid obesity which might be related to mechanical conditions but also to a higher load of metabolic comorbidities (potential mediators between obesity and mortality).

Overcrowding of the ICU facilities and resource rationing revealed the course of the disease and provided valuable information regarding the natural history of this condition. The main cause of death in patients who were not admitted to the ICU was hypoxemic respiratory failure or probable ARDS (although the Berlin definition of ARDS was not strictly fulfilled because they did not received a PEEP > 5 mmHg, the implications of this concept has been recently discussed by Tobin et al. [40] and might be irrelevant to the purpose of this study) unlike those patients who were admitted and died in the ICU in whom the cause of dead was primarily due to septic shock, followed by ARDS and multiorgan failure. Although the initial failure conditioning survival is the respiratory failure, in advanced stages (after providing initial ventilatory support) viral septic shock seems to prevail as the major event leading to death, this chronological distinction has not been dissected in previous reports in which all the patients who died received IMV and ICU admission [41–43]. As it has been previously suggested [43–45], the pathways leading to SARS-CoV-2 related death should be distinguished between those directly attributed the viral infection, those in which the infection partially contributed to the cause of death (i.e. nosocomial infections) and those unrelated to it, in this cohort the vast majority of deaths were directly related to SARS-CoV-2.

Besides the intrinsic limitations of a cohort study [46], ours has several additional ones. First, the population studied was limited to the metropolitan area of Mexico City, therefore extrapolation to areas with less population density (and less resource availability) might be inaccurate. Second, a considerable percentage of this cohort was transferred either to a convalescent center (due to clinical improvement) or to other hospitals with ICU bed availability (due to clinical deterioration and saturation of critical care areas of this tertiary care center) therefore the clinical endpoint of this subgroup is unknown and might have affected the analyzed data.

In conclusion this study represents a large prospective cohort exploring in-hospital mortality in COVID-19 pneumonia in Mexico.

Although unfortunate, this analysis reveals an unspoken problem in limited resource countries regarding supplies availability to handle health care challenges such as the SARS-CoV-2 pandemic. It has been stated through statistical modelling that high poverty indexes, lack of access to appropriate medical care and geographical location are major determinants for mortality among Mexican patients with SARS-CoV-2 infection [11]. To this data we may add the alarming situation of emergency department/critical care areas overcrowding and its impact in patient outcomes. Understanding and acknowledging this drawback might help forecast and prepare for future needs (particularly by reason of the prolonged plateau that is foreseen in this country). More data regarding outcomes in underdeveloped areas is needed to understand the scope and different scenarios of this pandemic in order to create strategies tailored for middle and low-income countries.

## Supporting information

S1 Fig Emergency department consults. (TIF)
