# Correction: In-hospital mortality from severe COVID-19 in a tertiary care center in Mexico City; causes of death, risk factors and the impact of hospital saturation

**DOI:** 10.1371/journal.pone.0269053

**Published:** 2022-05-23

**Authors:** Antonio Olivas-Martínez, José Luis Cárdenas-Fragoso, José Víctor Jiménez, Oscar Arturo Lozano-Cruz, Edgar Ortiz-Brizuela, Víctor Hugo Tovar-Méndez, Carla Medrano-Borromeo, Alejandra Martínez-Valenzuela, Carla Marina Román-Montes, Bernardo Martínez-Guerra, María Fernanda González-Lara, Thierry Hernandez-Gilsoul, Alfonso Gulias Herrero, Karla María Tamez-Torres, Eric Ochoa-Hein, Alfredo Ponce-de-León, Arturo Galindo-Fraga, David Kershenobich-Stalnikowitz, José Sifuentes-Osornio

The fourteenth author’s name is spelled incorrectly. The correct name is: Karla María Tamez-Torres.
