# Supplementary figures and images for: In-hospital mortality from severe COVID-19 in a tertiary care center in Mexico City; causes of death, risk factors and the impact of hospital saturation
Source: PLoS One. 2021 Feb 3;16(2):e0245772. doi: 10.1371/journal.pone.0245772 (PMC7857625; doi:10.1371/journal.pone.0245772)

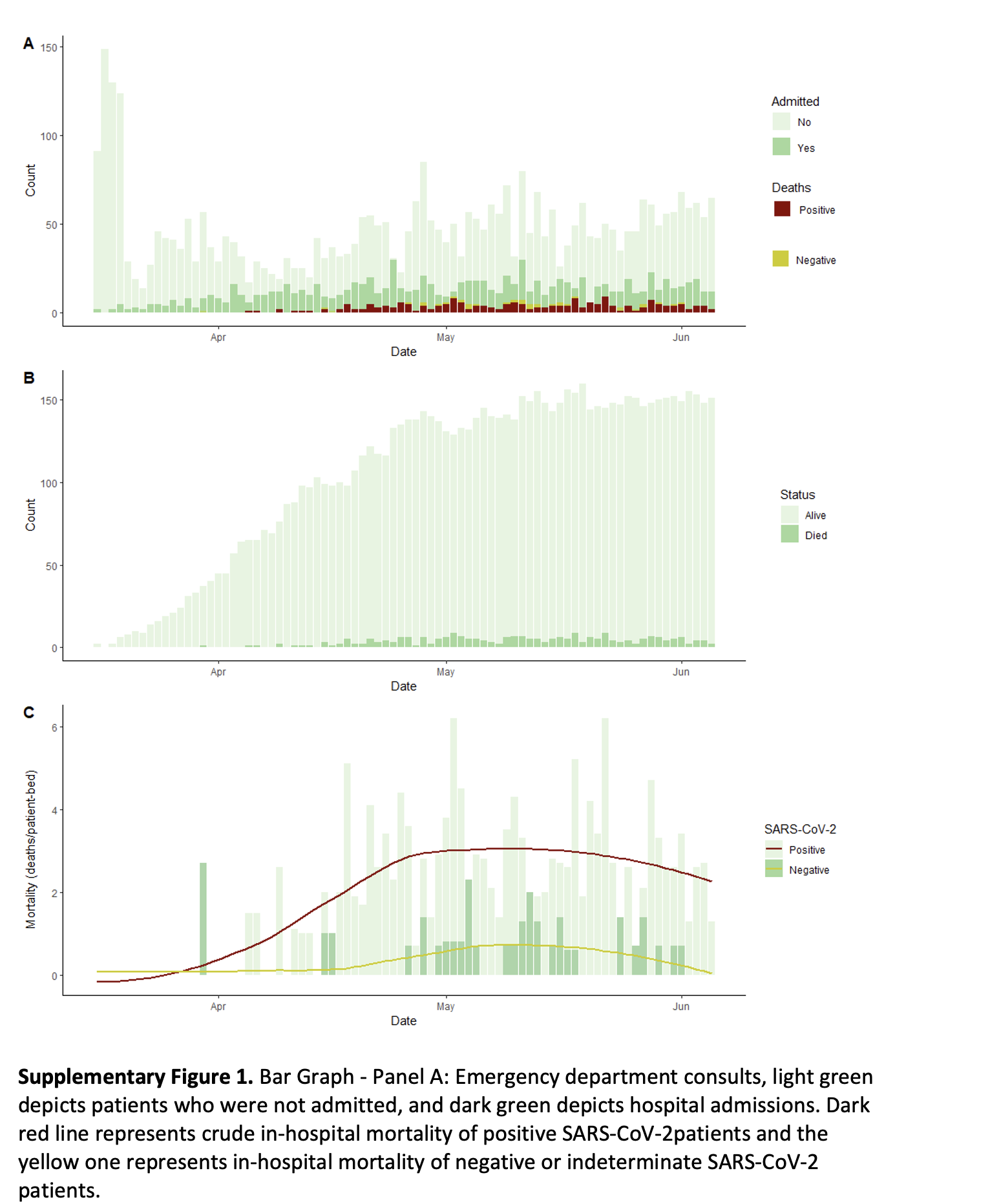

Supplement: S1 Fig — (TIF) [file pone.0245772.s001.tif]
